# Plant and microbial science and technology as cornerstones to Bioregenerative Life Support Systems in space

**DOI:** 10.1038/s41526-023-00317-9

**Published:** 2023-08-24

**Authors:** Veronica De Micco, Chiara Amitrano, Felice Mastroleo, Giovanna Aronne, Alberto Battistelli, Eugenie Carnero-Diaz, Stefania De Pascale, Gisela Detrell, Claude-Gilles Dussap, Ramon Ganigué, Øyvind Mejdell Jakobsen, Lucie Poulet, Rob Van Houdt, Cyprien Verseux, Siegfried E. Vlaeminck, Ronnie Willaert, Natalie Leys

**Affiliations:** 1 Department of Agricultural Sciences, University of Naples Federico II, via Università 100, 80055 Portici (NA), Italy; 2 Microbiology Unit, Nuclear Medical Applications, Belgian Nuclear Research Centre (SCK CEN), 2400 Mol, Belgium; 3 Istituto di Ricerca sugli Ecosistemi Terrestri Consiglio Nazionale delle Ricerche Viale Marconi 2, 05010 Porano (TR), Italy; 4 Institute of Systematic, Evolution, Biodiversity, Sorbonne University, National Museum of Natural History, CNRS, EPHE, UA, 45, rue Buffon CP50, 75005 Paris, France; 5 Institute of Space Systems, University of Stuttgart, Pfaffenwaldring 29, 70569 Stuttgart, Germany; 6 Université Clermont Auvergne, Clermont Auvergne INP, CNRS, Institut Pascal, F-63000 Clermont-Ferrand, France; 7 Center for Microbial Ecology and Technology, Ghent University, Coupure Links 653, 9000 Gent, Belgium; 8 Centre for Interdisciplinary Research in Space (CIRiS), NTNU Social Research, Trondheim, Norway; 9 Center of Applied Space Technology and Microgravity (ZARM), University of Bremen, 28359 Bremen, Germany; 10 Research Group of Sustainable Energy, Air and Water Technology, University of Antwerp, 2020 Antwerpen, Belgium; 11 Research Groups NAMI and NANO, Vrije Universiteit Brussel, Pleinlaan 2, 1050 Brussels, Belgium

**Keywords:** Plant sciences, Microbiology

## Abstract

Long-term human space exploration missions require environmental control and closed Life Support Systems (LSS) capable of producing and recycling resources, thus fulfilling all the essential metabolic needs for human survival in harsh space environments, both during travel and on orbital/planetary stations. This will become increasingly necessary as missions reach farther away from Earth, thereby limiting the technical and economic feasibility of resupplying resources from Earth. Further incorporation of biological elements into state-of-the-art (mostly abiotic) LSS, leading to bioregenerative LSS (BLSS), is needed for additional resource recovery, food production, and waste treatment solutions, and to enable more self-sustainable missions to the Moon and Mars. There is a whole suite of functions crucial to sustain human presence in Low Earth Orbit (LEO) and successful settlement on Moon or Mars such as environmental control, air regeneration, waste management, water supply, food production, cabin/habitat pressurization, radiation protection, energy supply, and means for transportation, communication, and recreation. In this paper, we focus on air, water and food production, and waste management, and address some aspects of radiation protection and recreation. We briefly discuss existing knowledge, highlight open gaps, and propose possible future experiments in the short-, medium-, and long-term to achieve the targets of crewed space exploration also leading to possible benefits on Earth.

## Mimicking nature: towards an integrated view of life in BLSS

**Fig. 1:**
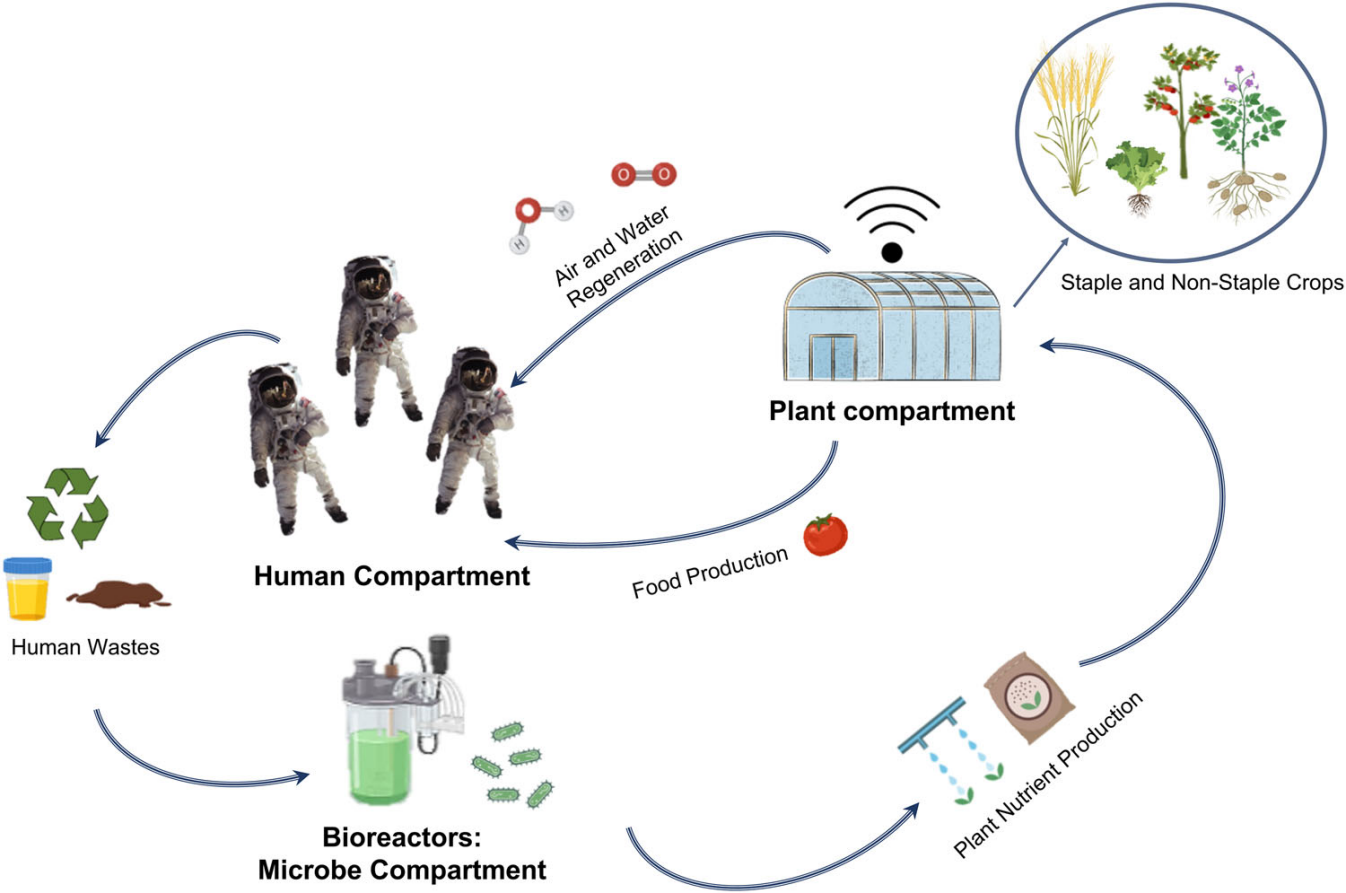
Schematic example of Bioregenerative Life Support System including the three main compartments: (i) the human compartment, (ii) the microbe compartment, and (iii) the plant compartment. The focus is on the regeneration of resources and the interconnected compartments based on organisms’ wastes from each compartment representing inputs for the other compartments.

The concept of Bioregenerative Life Support Systems (BLSS), also called Closed (or Controlled) Ecological Life Support Systems (CELSS), has been explored since the beginning of the human space exploration era in the 1960s^1^. A closed and semi-closed loop BLSS is based on the concept of ecological networks where several levels of trophic connections guarantee biomass cycling in food webs. Thus, a BLSS is made of several interconnected compartments based on organisms whose wastes represent the vital resources for the other compartments (Fig. 1). These systems comprise three main types of compartments: biological ‘producers’ (e.g., plants, microalgae, photosynthetic bacteria), ‘consumers’ (i.e., crew), and waste ‘degraders and recyclers’ (e.g., fermentative and nitrifying bacteria). Several alternative biological elements have been proposed over the years^2^. For example, animal compartments (e.g., with insects, fish) have been proposed to provide additional proteins^3^. Several large-scale ground-based demonstrators have tested closed-loop BLSS with humans in the loop, such as BIOS-1, 2, 3, and 3 M in Russia, Biosphere 2 in the USA, the Closed Ecology Experiment Facility (CEEF) in Japan, and Lunar Palace 1 in China. Other facilities in Antarctica have tested independent BLSS functions like gray water recycling at the Concordia station or food production within EDEN ISS (International Space Station) Mobile Test Facility at Neumayer Station III. Moreover, within the NASA Lunar-Mars Life Support System Test Project, a growth chamber contributed to the air revitalization and food requirements of a crew of four for 91 days^4^. Other facilities have enabled analog missions testing compartments or parts of them with various levels of complexity, such as MARS500 and SIRIUS in Russia, HERA and LMLSTP at NASA’s JSC, KSC’s Biomass Production Chamber, MDRS in Utah and HI-SEAS in Hawaii, USA^4–6^. These ground-based demonstrators have been used to test specific technologies for controlled cultivation chambers, food production systems, and biological waste management. Some of these test facilities have also been used to evaluate the impact of confinement on isolated crews in terms of physiological and psychological issues, as well as to evaluate the possible mitigation effect of the presence of plants (e.g., access to fresh food, gardening for recreation)^7,8^.

International space agencies have successfully developed regenerative systems for the recovery and purification of air and water for their crew, during missions in Low Earth Orbit (LEO) (e.g., in the Space Shuttle, the Soyuz, and the Salyut, ISS, and Chinese space stations). Currently, from a European perspective, the European Space Agency (ESA) does not have its own integrated BLSS ground test facility able to host a human crew and still relies on other international partners and collaborative projects (e.g., testing of water purification systems at Concordia in Antarctica). However, over the last three decades, contributing countries have invested through ESA in the Micro-Ecological Life Support System Alternative Program (MELiSSA), which led among others to the construction of a MELiSSA pilot plant in Spain (MPP) and a plant characterization unit in Italy (PaCMan)^9^. Both facilities are aimed at designing and testing a closed-loop system providing oxygen, potable water, and fresh food, by recycling organic and inorganic wastes. However, the MPP is a ground demonstrator of multiple compartments of the MELiSSA loop and their connection, while PaCMan is focused on fundamental biological experiments on plants in a closed chamber.

To date, bioreactors or plant cultivation components of BLSS have already been tested in LEO, onboard FOTON and the International Space Station (ISS), as a proof of concept^10,11^, demonstrating it is feasible to run a bioreactor or grow plants in LEO. However, these tests were typically done on single biological systems in small scales (i.e., less than 100 mL or 0.2 m²), with low overall yield, over short durations, and with significant crew activity involved. Future BLSS compartments supporting the “consumers/astronauts” compartment will need to be scaled up and optimized for efficiency, robustness, autonomy, remote control, and integration into complex habitats. Complete integration of all compartments in ground demonstration facilities is the first logical step to these endeavors. Similar systems with the addition of a few pressurized modules could then be used on the Moon and, eventually, the Lunar surface could be used as a testbed for future Mars missions, necessary to guarantee the outpost’s autonomy^12^. In addition to the challenges faced when fully integrating all compartments on Earth, the impacts of space environmental conditions (such as reduced gravity, increased ionizing radiation, lower atmospheric pressure, regolith dust, different atmospheric composition, and magnetic fields) on the biological components and processes of BLSS need to be taken into account as this could impact their efficiency and the input/output balance among the interconnected compartments^13,14^. In this paper, we focus on the two main groups of BLSS organisms, namely plants, and microbes. We will briefly highlight what is known and what are the existing knowledge gaps that are relevant to the design of BLSS. Moreover, a section is dedicated to the design and realization of BLSS, including the possible use of biomaterials directly produced in space. We then present possible future research questions, and technological challenges to face in the short, medium, and long term which must be addressed to achieve the targets of life support in human space exploration.

## The higher plant compartment

Plants are the primary food producers for humans on Earth and have the potential to accomplish the same task in space. Furthermore, by consuming carbon dioxide and producing oxygen through photosynthesis, purifying water from the collection of transpired water, and having a role in waste recycling, they are well-suited for the regeneration of resources^15^. Although regeneration of resources in short-term missions would be a “nice-to-have” requirement, it becomes a “must-have” in long-term missions where resupply from Earth would not be feasible and where initial launch mass to carry all consumables would be prohibitive.

It might be questioned that air and water regeneration can be achieved with other photosynthetic organisms or physicochemical processes. However, it is a fact that the cultivation of higher plants provides an added value in terms of food production in the case of crops, because currently, the only way to produce food in space is via biotransformation, that is not feasible with physicochemical processes. Indeed, from a nutritional and functional point of view, the integration of astronauts’ diet with plant-derived, highly nutritious, fresh food can help counteract diseases (both physiological and psychological) induced by the stressful space environment^16,17^. Currently in space, only pre-packaged food is used (apart from bonus fresh food in the frame of selected experiments), and it tends to lose nutrients and vitamins over time. Cooper et al. (2017)^18^ found that, in space food, Vitamin C and B1 degrade to inadequate concentrations within 3 years at 21 °C storage, while vitamins A, B6, and B12 decline but sufficient concentrations remain after 3 years. For a Martian mission, food shipped from the Earth may have to be stored for several years, likely resulting in low vitamin content at the time of consumption. Plants may also provide non-nutritional benefits, such as psychological support against conditions of isolation, acting as emotional supporters in a sort of “horticultural therapy”^19^.

### The mission scenario

Taking for granted the need to cultivate plants in space, the BLSS design, including the choice of the species/cultivars and the cultivation systems, should be tuned to the mission scenario and its duration. Indeed, different mission scenario are differently influenced by the environmental factors and differently influence the possibility of resupplying resources from Earth^20^.

For short-duration human missions, such as those on Earth-orbiting platforms (LEO), crop production should be directed towards fast-growing species, occupying minimum volumes, providing high nutritive values, such as leafy greens (e.g., lettuce, kale), microgreens or sprouts, dwarf cultivars of horticultural crops (e.g., tomato). The concept of a vegetable production unit also defined as a “salad machine” to integrate astronauts’ diets, has been proposed by researchers since the early 90’^21,22^. These species will complement the astronauts’ diet (which is still dependent on Earth supply in such mission class) and, being rich in nutraceuticals (such as antioxidants and prebiotics), will help to strengthen the physiological defenses of the astronauts’ bodies against the diseases induced by the exposure to space factors^23^. This type of plant growth facility would not contribute substantially to resource recycling (especially in the case of sprouts that are still in too early a stage of development to have an active photosynthetic activity) but require only minimal inputs, such as low-energy, small growing area, short-time, and basic technological integration with the rest of the facilities^24,25^. However, it must be taken into account that crop systems like microgreens need a high number of seeds, which can represent a significant upload mass in short-duration missions.

For long-duration missions and the realization of stable planetary outposts, staple crops (e.g., wheat, potato, rice, soy) must be included to provide the carbohydrates, proteins, and fats of the basic diet. Also, several vegetables and fruits with longer growth cycles (~100 days, e.g., tomato, peppers, beans, and berries) can be included^26^. In this scenario, crops are selected based on their nutritional value, requirements of resources (e.g., water, nutrients, light), edible/waste biomass ratio, storage requirements, and waste treatment requirements^27^. The contribution of plants to resource recycling in this case will be substantial and the cultivation will require a large growing area per astronaut and a deep integration with the rest of the system and subsystems of the platform. In the case of long-duration missions, seed production becomes a necessity, too. The *seed-to-fruit* and *seed-to-seed* cycles are delicate phases in the plant reproductive cycle^28^. Experiments in simulated microgravity demonstrated the possible occurrence of aberrations in pollen tube development^29,30^. Therefore, the achievement of these cycles can take a significant amount of time and resources. In both short- and long-duration mission scenarios, acclimation and adaptation to the space environmental factors will be crucial criteria for species and cultivar selection.

### Impact of the space factors on cultivation requirements

On Earth, plants need to invest resources to build leaves capable of converting carbon into biomass by enhancing photosynthetic carbon gain and controlling water losses through evapotranspiration. Photosynthetic and hydraulic performance is mediated by structural and physiological traits, which have evolved over millions of years in the presence of “Earth” factors^31,32^, while they can be severely affected by space factors^33^. Among these, there are those factors present on Earth but at different levels (e.g., temperature, light, pressure, atmosphere composition), new factors (e.g., altered gravity and ionizing radiation), and secondary factors such as physical processes altered by new factors (e.g., lack of buoyancy-driven convection)^34^. For example, environmental factors, especially humidity, and temperature, interact with pollen development and functionality of candidate crop species for space cultivation^35^. Together with an efficient photosynthetic apparatus and hydraulic system (e.g., efficient transportation and distribution of water through the xylem and stomatal control), efficient plant growth and reproduction relies on many other complex processes such as cell proliferation, organogenesis, sporogenesis, and gametogenesis, controlled at different levels (e.g., molecular, cellular, structural, physiological, and biochemical) by intrinsic and environmental factors. The advancement of knowledge in the effect of space factors on fundamental biological processes becomes crucial because the alteration of such processes has a deep impact on the requirements for the design of BLSS. For example, the alteration of the photosynthetic/hydraulic coordination would have a direct impact on O_2_ production, changing the existing balance with the crew compartment and thus requiring different set-up for the environmental control. Plant reproduction in space is essential to make missions independent from Earth; seed supply and experiments are needed to advance knowledge to ensure successful reproduction in space^36,37^. Maximizing resources and nutritive value, while minimizing wastes and ensuring seed-to-seed production requires the selection of genotypes that can sustain space conditions, as well as the accurate and reliable control of environmental and cultivation conditions. This progressive selection of crop species for space can be tracked using the Crop Readiness Level (CRL), analogous to the Technology Readiness Level (TRL). First introduced about 20 years ago, the CRL was reproposed in 2019 by Romeyn et al.^38^ for ISS and early LEO testing. It aims to track the testing of different crop species for their use in the space environment. Like the TRL, the CRL is based on a 1–9 scale and assigns “1” to the identification of a potential crop and “9” to the final stage of growing a crop in space. In the middle, scalar tests under various controlled conditions with different endpoints should be performed. The main target of fundamental plant biology experiments in space has been the study of the impact of microgravity and ionizing radiation on plant growth and physiological processes (e.g., hormone signaling, cell differentiation, tropic responses, and reproductive aspects). From these experiments, it could be concluded that LEO microgravity does not hinder plant growth, at least not directly^39,40^. Many of the aberrations in plant growth found in early space experiments, were later found to be caused indirectly by microgravity, due to the lack of buoyancy-driven convection on the air which alters gas exchange and water fluxes in the absence of a fine environmental control^41,42^. This indicates again that the agricultural system and the fine control of the plant growth compartment is key to achieving good food production in quantitative and qualitative terms. Early experiments have demonstrated changes in the food quality of vegetables produced in space^43^, but more tests are needed especially increasing the control of sample stability during Earth re-entry.

The effects of space radiation have been much less studied, probably because all the experiments so far conducted in LEO, often inside the LEO facilities, and are performed with short exposure, leading to doses that are too low to elicit severe modifications in microbes or higher plants, which are generally more resistant than mammals^44^. Moreover, most available information derives from ground-based studies where the target of acute irradiation was dry seeds which are characterized by the highest resistance to abiotic and biotic factors^13^. A true assessment of radiation-induced alterations during plant development can be only achieved by analyzing the effect of real space radiation on actively growing tissues. However, the hardware currently available to expose organisms directly to the space environment, without significant shielding (e.g., BIOPAN, EXPOSE), are not suited for the active growth of higher plants because of the reduced volumes available and the lack of specific environmental control.

### The use of external resources in BLSS

Another crucial point is the cultivation system which depends on many technical factors, also depending on the mission scenario, which dictates the requirements in terms of mass and energy budget and therefore of all the materials to be used. So far, plant experiments in space have mainly been done with a granular media or gel- and mat-like substrate to hold the seeds/plants in place and controlled release fertilizer pellets with the addition of water^10^. However, in an outpost, plant cultivation could also rely on In Situ Resources Utilization (ISRU) based technologies. This includes the use of Lunar and Martian regolith, treated to render them biocompatible (non-toxic) with fertilizers, amendments, and/or treated crew wastes to add organic material to the barren inorganic minerals^45,46^. Some baseline studies of plant growth in regolith simulants have been done and are ongoing. However, none of the available simulants seems to cover all relevant features, such as exact mineral composition, redox chemistry, or grain shape. Moreover, there are also operational challenges related to the use of regolith in altered gravity, mainly related to dust contamination inside the settlements, airlock events and above all to water drainage which directly influences plant hydraulics. For the latter, one solution proposed to counteract the reduced gravity effect, was to increase the medium particle size above 1 mm and narrow their distribution^47^.

### Knowledge gaps for the plant compartment

**Fig. 2:**
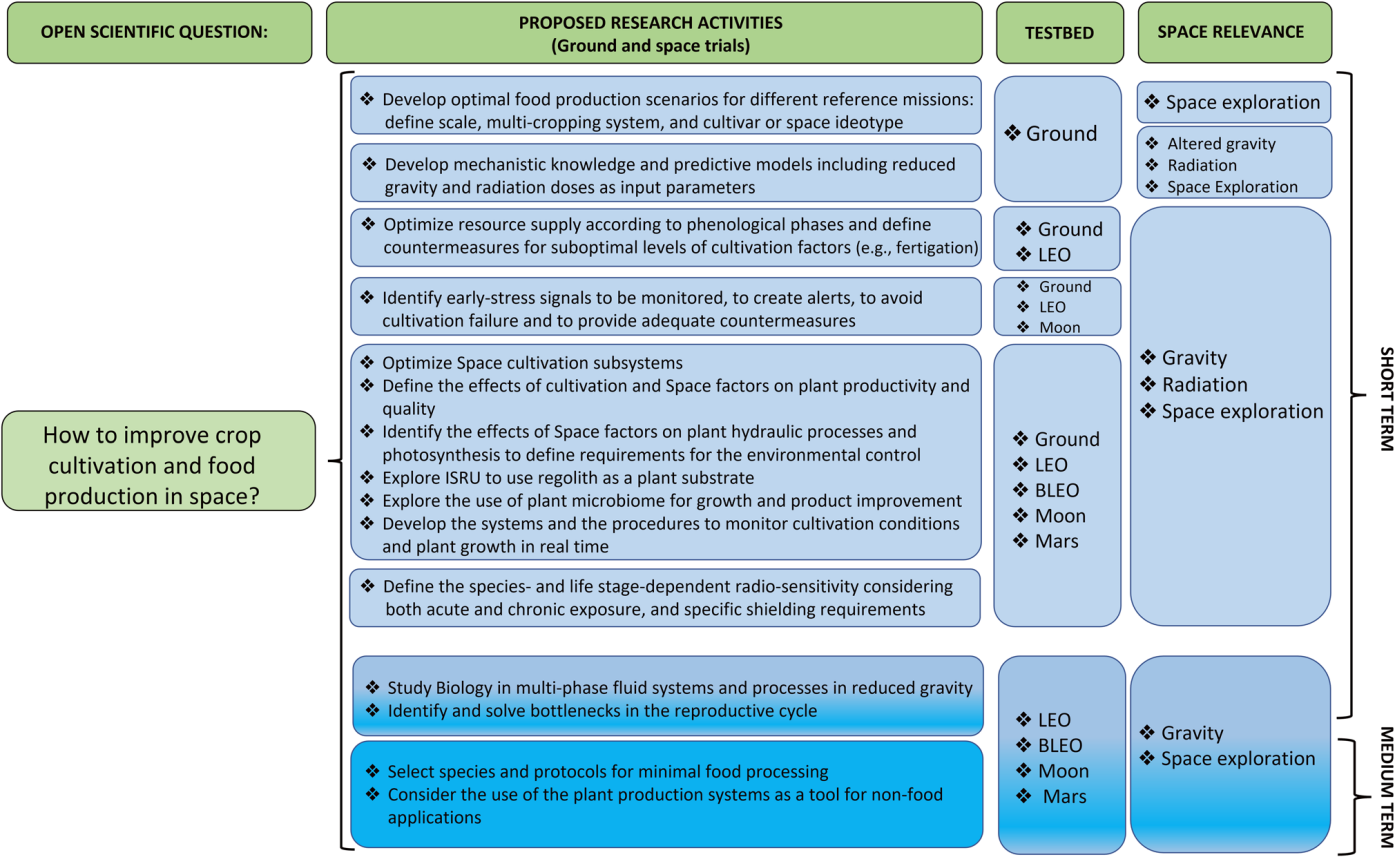
Summary of possible sub-goals within the main goal of improving crop cultivation and food production in space in the timeframe of 2022–2030 and beyond (short-term, 2022–2024 and medium-term, 2024–2030) using the ISS as a primary platform. Other research platforms such as the ground, Moon, Mars, and BLEO (Beyond LEO) are also included. BLEO is referred to long-term missions beyond LEO, also involving only traveling/transit without the permanence on a planetary station. They represent both the basis for research on ISS and future research activities post-ISS (e.g., the Moon-orbiting GATEWAY).

With the final goal to allow human habitability in space, a series of knowledge gaps can be identified that culminate in the main open scientific question, namely, how to improve crop cultivation and food production in space (Fig. 2). The identification of knowledge gaps requires a preliminary definition of the optimal food production scenarios corresponding to reference mission scenarios, which define mission constraints and opportunities. The definition of such optimal scenarios is itself a gap where the first steps are the definition of the scale of the plant growth compartment (related to crew size and available space), the duration of the mission, energy resource availabilities, the possibility to use external resources and multi-cropping systems. There is also the need to improve species and cultivar selection, including ideotype breeding for fitting the specific different reference missions. Although extensive information is available on both plant biology and horticulture in space, there remains a need to define the main trends, independently from the different experimental conditions used. The aim is to develop mechanistic knowledge and predictive models of plant growth in space plant compartments, including reduced gravity and radiation as input parameters^48^. Ground-based research and experiments in LEO should be targeted to optimize resource supply according to phenological phases and to define countermeasures in case of reaching suboptimal levels. The monitoring to identify plant early-stress signals is fundamental to create alerts, avoid cultivation failure, and provide adequate countermeasures in case suboptimal (off-nominal) conditions occur^49^.

A set of gaps needs to be filled to optimize space cultivation subsystems, which include knowledge gaps in plant biology and agro-technologies (Fig. 2). Filling these knowledge gaps will allow defining species-specific protocols and procedures to optimize resource-use-efficiency for plant cultivation in BLSS (also in the presence of space factors), based on the identification of growth requirements, which change according to developmental stage (seed, sprout, seedling, adult plant) or phenological phase (vegetative growth, flowering, fruiting). Parthenocarpy, or seedless fruit development, can be useful to develop fruits under environmental conditions that are unfavorable for successful pollination and fertilization^50^. Asexual reproduction can ensure the regeneration of food resources and stable nutritional value, while sexual reproduction can guarantee a higher propagation coefficient and lower storage and transportation costs^51^.

Therefore, there is a need to test and identify which are the best and most sustainable (1) substrates (to promote *ISRU*), (2) water and nutrient delivery systems, (3) atmosphere management systems, and (4) illumination systems. More specifically, it is necessary to understand the impact of direct space factors and other factors (e.g., limited volumes) on water and nutrient uptake, as well as the effect of different cultivation systems and usages on the long-term modifications of growth substrates and water delivery dynamics. It is important to consider atmosphere management, i.e., to understand the direct effect of relative humidity, ventilation, and atmosphere composition on plant morphogenesis, physiological processes (especially regarding the coordination of plant hydraulics and photosynthesis), productivity, yield, and quality (nutritional value and safety) of the produced food. Concerning the illumination system, it is necessary to understand and optimize the effect of light quantity and quality on plant morphogenesis, physiological processes, productivity, and nutritional values. Ultimately, defining the environmental and cultivation requirements, which change according to not only species/ cultivar but also to the life stage and phenological phase, becomes necessary.

There is also a need to unravel the impact of the effect of space and cultivation factors, alone or in interaction, especially on the coordination between plant hydraulics and photosynthesis in the soil/substrate-plant-atmosphere continuum by considering the plant as a whole. To achieve these purposes, it appears necessary to perform more morpho-physiology and molecular biology tests (to investigate genome stability and metabolism regulation) on crop species (in addition to what has been done on model plants such as *Arabidopsis thaliana*) and to differentiate between plants’ acclimation and adaptation, especially when considering the need to guarantee viable seed production for multiple generations. To guarantee the latter point, there is a need to identify bottlenecks in the reproductive cycle by focusing on the whole cycle or on specific phases of the seed-to-seed process (e.g., flower development, pollen viability, fertilization, embryogenesis) to define environmental requirements and technical solutions to overcome these constraints.

Specific actions to fill the gaps to be addressed in plant biology to support the design of BLSS are:

- Improve the knowledge on root growth orientation, mainly focusing on interactions of multiple tropisms, to give insights for the design of plant growth chambers.
- Identify the effects of multiple space factors (e.g., altered gravity and radiation) on the regeneration capacity of plants both by cloning and by reproduction.
- Study biology in multi-phase fluid systems and processes in altered gravity, including liquid/liquid, liquid/gas, and liquid/solid, with active biological production of compounds to understand the effect of altered gravity on:

1. plant hydraulics and gas exchange, to define requirements for the environmental control and monitoring of the plant growth chambers, with specific emphasis on humidity control, airflow, and atmosphere composition.
2. water and solute transport (root absorption, xylem, and phloem sap flows) to improve cultivation substrates and water/nutrient delivery systems.

- Define the shielding requirements considering the different radio-sensitivity of different species, cultivars, and life stage-dependent sensitivity, also considering the possible hormetic effects (e.g., stimulation of different biological processes occurring when organisms are subjected to irradiation with low doses).
  Other knowledge gaps pertain to interactions with other compartments. Indeed, it is fundamental to assess the role of the microbiome and the interactions between plants and beneficial/pathogenic microorganisms in space conditions. Upstream microbial processes in the closed loop of BLSS will likely result in dynamic and non-optimal crop cultivation conditions (nutrient levels/ratios); it is, therefore, necessary to improve our knowledge of the plants’ vulnerabilities and thresholds and to identify means of mitigation.
  From a technological point of view, it is crucial to develop systems and procedures to monitor cultivation conditions and plant growth in real-time and to adopt countermeasures in case of alerts. This includes the development of:
- Miniaturized sensors for remote control, monitoring, and modeling of plant growth to forecast plant productivity in case of anomalies in the different cultivation subsystems, and for early detection of plant stress symptoms and diseases (e.g., hyperspectral and multispectral imaging).
- Miniaturized sensors for monitoring the cultivation conditions. In a closed loop, with dynamic upstream processes, e.g., monitoring chemical and microbial water quality is very important.
- Procedures for real-time adjustment through remote control of environmental/cultivation/growth parameters.

In the species/cultivar selection, apart from the criteria related to the nutritional value and health-promoting molecules (which should be preferred), cultivation requirements, and resistance to space factors, other criteria should be considered. In a long-term vision, the selection should consider species to be destined for minimal food processing, also setting-up protocols, and menus for the minimal process of food, while respecting the food safety aspects. It is understood that this may not be possible for all crops, and some process-intensive staple crops will still need to be included (e.g., wheat, potato, rice). Some^52^ have already attempted to develop a menu for Advanced Life Support (ALS) based on the crops list of the Closed Ecology Experiment Facilities (CEEF). Those recipes were evaluated using a few indexes including nutritional contents, acceptability, fresh weight of ingredients, and necessary cultivation area of each.

The integration of all gained knowledge of past and future experiments should culminate in the analysis of how environmental/space and cultivation factors can be harmonized to improve plant-based food production in space maximizing the nutritional quality of plant-derived food while reducing anti-nutritional factors.

## Microbe compartment

Microbial biotechnology targets the design, engineering, and control of microbial bioprocesses toward desired end products. Microbial bioprocesses are widely applied on Earth, from wastewater treatment and organic waste processing to food production (e.g., dough, beer, cheese, yogurt), industrial biotechnology and drug production. Although currently nearly absent in space, microbial biotechnology is essential for resource recovery (i.e., ‘closing the loop’), allowing for more resource-efficient air revitalization, water reuse, waste treatment, food production, and production of N_2_ as inert atmosphere gas in BLSS. For future missions to the Moon and Mars, additional microbial biotechnological applications must be explored. Little has been done so far on microbial or microbially-assisted food production and fermentation processes, probiotics and nutraceuticals, and pharmabiotics (e.g., antibiotics) production in space. One could investigate the development of onboard ‘DIY’ (batch) cultivation facilities and kits for fermentation or food production (e.g., bread dough and baking) that are safe and suitable for direct harvest and consumption, and require minimal food processing, nevertheless providing the crew with health-promoting food products.

One of the most advanced concepts for bioprocesses in space involves microalgal photobioreactors. They have been well studied on Earth, with over 30 years of data and knowledge documenting high production rates for many species^40^. Earth applications are emerging at full scale and include water purification, CO_2_ capture, and conversion, as well as the production of biomass for proteinaceous food supplements, biofuels, pigments, and other outputs. Indeed, the generated biomass can be recycled or otherwise valorized. Approaches under investigation include: its use as a nutrient source for other plant- or microorganism-based processes^53^; its incorporation into 3D-printing feedstock^54^; and, in the case of edible species, its consumption by the crew as a protein-rich dietary supplement^55^. Many BLSS under development have indeed selected microalgae, including cyanobacteria, for efficient CO_2_ removal and O_2_ production, and as food supplements. Microalgal processes and bioreactors have been taken up from the start of BLSS development and have been studied in space, while other bioprocesses (e.g., nitrification for urine treatment, anaerobic fermentation for waste degradation, air biofilters and off-gas treatment, regolith weathering for metal mining and nutrient mobilization) have been much less explored^56,57^.

To our knowledge, only two microalgal photobioreactors have been sent to space and only one was successfully run for several weeks inside the ISS^11^. The latter was only 50 mL. It was run in batch mode and focused on biomass production for cellular biology, molecular biology, and biochemistry research. The photobioreactor mechanistic model developed for O_2_ production fitted the experimental data obtained on this ISS experiment^11^. Little knowledge has been generated in space conditions on product conversion and biochemical end products (as would be needed, e.g., waste degradation, water purification, or fermentation), or on the kinetics of bioprocesses in space. On-line monitoring was often incompatible with the flight hardware (i.e., mass, size, and energy restrictions) or deemed too expensive. Therefore, real-time bioprocess control and data transfer remained very limited. In addition, the evolution of the bioreactor’s microbial strain or community over multiple generations in space, and consequently the potential drift in process efficiency or products, also remains to be explored.

Bioreactors confine bioprocesses and enable their control, independently of natural environmental conditions. Depending on their purpose, the aim is either to maximize growth (e.g., production of edible biomass) or to increase their performance in terms of degrading waste products. A great challenge in space bioreactors is to impose and regulate the same process conditions in Space as normally done on Earth (i.e., light, nutrition, temperature, pH, and water availability), considering strict safety issues, as well as mass, power, and volume constraints. Another challenge concerns the integration of biological and physicochemical components and in this sense the experiment Photobioreactor at the Life Support Rack (PBR@LSR) was launched to the ISS in the second quarter of 2019. The objective was to prove the feasibility of xenic long-term cultivation of microalgae (*Chlorella vulgaris*) under space conditions and to demonstrate for the first time the technology and performance of a hybrid life support system (combining physicochemical and biotechnological components)^58^. Finally, the gravity- and radiation-related phenomena in microbial bioreactors in space and their impact on biology remain to be thoroughly investigated.

### Knowledge gaps for the microbe compartment

**Fig. 3:**
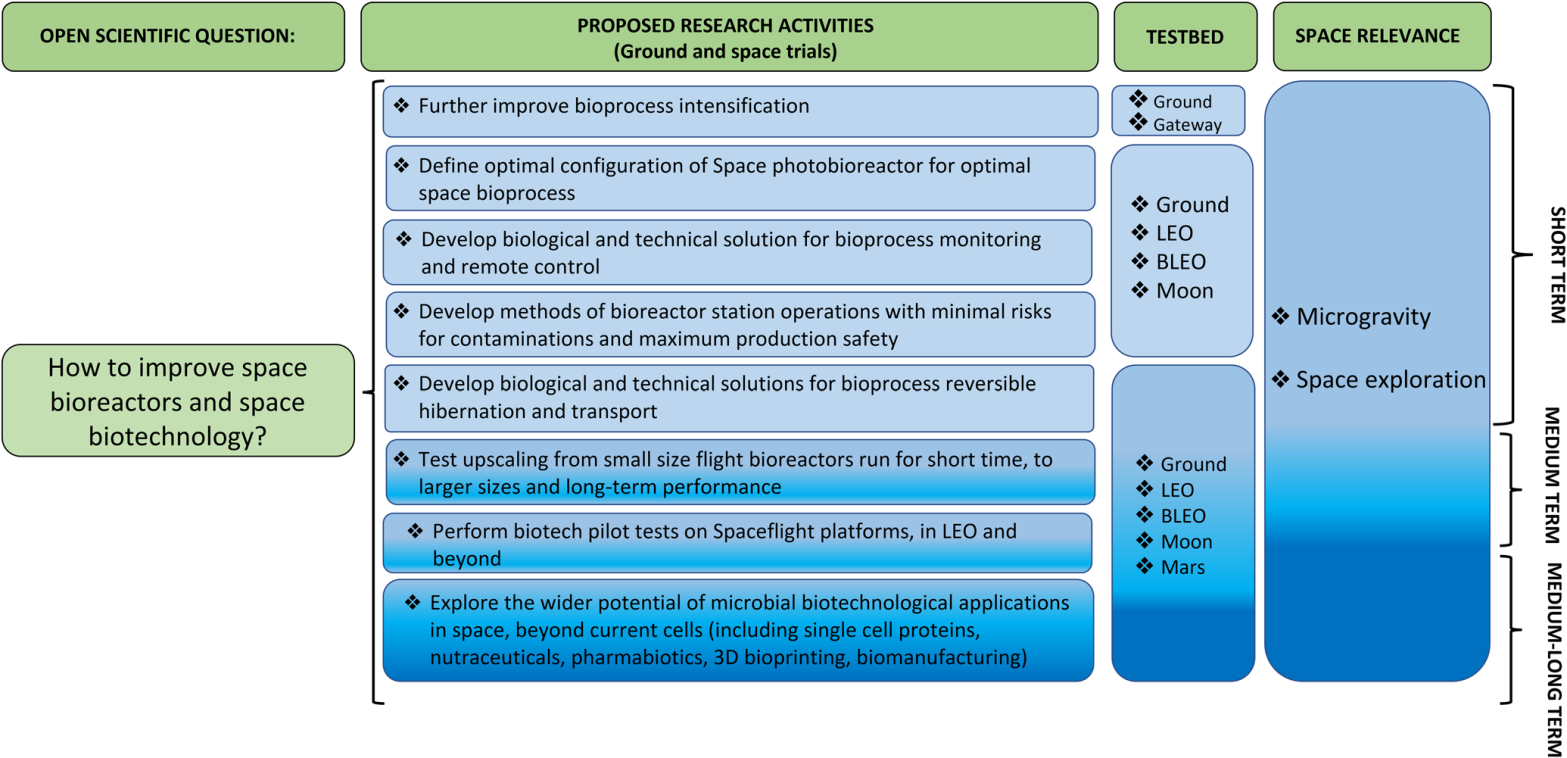
Summary of possible sub-goals within the main goal of improving space bioreactors and space biotechnology in the timeframe of 2022–2030 and beyond (short-term, 2022–2024; medium-term, 2024–2030; long-term, beyond 2030) using the ISS as the primary platform. Other research platforms such as the ground, Moon, Mars, and BLEO are also included. BLEO is referred to long-term missions beyond LEO, also involving only traveling/transit without the permanence on a planetary station. They represent both the basis for research on ISS and future research activities post-ISS (e.g., the Moon-orbiting GATEWAY).

With the final goal to support human space habitability, a series of knowledge gaps can be identified that culminate in the main open scientific question, namely, how to design effective space bioreactors (Fig. 3). The following actions and open issues need to be considered for space bioscience engineering and space biotechnology:

*Individual bioreactors/bioprocesses:*

- Develop adequate solutions (and technologies) for storage, transport, activation, and shutdown of microbial bioprocesses in space conditions accounting for altered gravity and increased ionizing radiation.
- Further intensify bioprocesses, and miniaturize bioreactors, for space missions implying highly constrained masses, volumes, and resources (e.g., implement/develop high-cell density processes/reactors; increase mass transport by mechanical mixing or pumping, potentially increasing shear stress; improve interphase mass exchange rates, e.g., gas-liquid exchange using membrane technologies).
- Define the best configuration for space photobioreactors and harvesting systems on a space station or outpost. It will be worth investigating whether biology (e.g., microorganism growth kinetics and development rate) and bioprocesses would benefit from countermeasures including artificial (hypo-) gravity, magnetic fields, and artificial or natural light.
- Develop adequate solutions (and technologies) for full remote biomonitoring and bioprocess control.
- Develop non-invasive and biocompatible sensors and analytical equipment, compatible with space conditions, performing in situ measurements of physiological transport (e.g., water and nutrients) and exchange (e.g., gases such as O_2_ and CO_2_) in a bioreactor culture.
- Characterize and understand phase separation (gas-liquid, liquid-liquid) and mixing, including determination and prediction of heterogeneities in reacting volumes, and the kinetics and stoichiometry for different configurations. Biomass harvesting and solid-liquid separation while keeping biomass safe for use with the best nutritional quality are also crucial steps to keep in mind.
- Determine microbial and chemical contamination/spoilage risks on bioreactor operations and products, and define storage and logistics of supplies (utensils, water, food, etc.) for spaceflight.

*Interconnected bioreactors/bioprocesses:*

- When various bioprocesses and bioreactors are coupled for circular loop closure and maintained operational over a long period, additional challenges manifest, related to:
  - How to prevent (cross)-contamination when coupling axenic processes?
  - How to prevent potential detrimental metabolites or (microbial) cell cross-talking (quorum sensing molecules) between interconnected bioreactors?
  - How to control the loop and how to deal with potential operational issues/failures of some of its elements?
- Develop ad hoc experiments and models to assess the bioprocesses at different scales in size and in time, depending on the different mission scenarios.

Current bioreactor experiments and tests have so far been run for short times (a few days or weeks). If those systems are going to be part of a BLSS, they will have to work for long periods reliably. The issue pertained to long-term performances must be addressed, including both technical and biological activities:

- Explore the usability (functionality and stability) of a variety of ground-based validated microbes and communities (including genetically engineered or otherwise synthetic ones) and identify the most suitable candidates for pre- and probiotic, nutraceutical, or medicine production in space, as protective or therapeutic countermeasures for radiation protection, healthy gut microbiome, digestion, skin and wound treatment, periodontal (mouth and tooth) health.
- Assess the potential of 3D food printing and other future food products from microbial and fungal sources.
- Further investigate biogenic *ISRU* processes, such as bioleaching (e.g., extraction of rare Earth elements) and biomineralization (e.g., biology-based bricks, reduction of regolith toxicity or dust via mineralization). This includes understanding microbe-mineral interactions using collected Moon or Mars samples and/or representative simulations under space conditions.
- Next to the ISS, exploit other LEO spaceflight platforms and opportunities as well as Lunar landers or rovers as a testbed for miniaturized components of LSS bioprocesses, to assess the effect of space environmental factors on the bio(techno)logical performances.

## Key elements for the design of a BLSS

The design of a BLSS is highly challenging and although several potential designs exist, high loop closure remains to be demonstrated, first with Earth demonstrators and then in space.

The driving elements for the design of an ideal BLSS are:

- Reliable control of atmosphere composition according to the requirements of the different sub-compartments.
- Sufficient and reliable humidity control, and maximum recovery of water into potable water.
- Production of safe food with high nutritional value, with minimal resource requirements, and maximum harvest index (ratio of edible to total biomass).
- Efficient management (e.g., containment for biosafety) and maximum re-conversion of wastes, CO_2_, and minerals, into resources for air revitalization (e.g., O_2_, N_2_) or crop production (e.g., NPK fertilizers), in a minimum number of simplified recycling steps, with minimal resource consumption.
- Using well-characterized, reliable, and safe organisms and communities, which preferably possess a certain degree of ‘space robustness’.
- Maintenance of a bio-safe and healthy habitat environment (kept free from waste, microbial, and chemical hazards).
- Small and lightweight, and easy to handle, self-regulating operational units (e.g., bioreactors, plant growth chambers) adapted for spaceflight (in case of orbital stations or space transit vehicles).
- Possible utilization of resources available in situ (including waste).
- Robust and effective mechanistic models of each compartment and the BLSS as a whole to predict and anticipate potential failures, enable robust and reliable control, and allow for system design evaluation and comparison.

Any BLSS design will require a smart combination of multiple organisms and bioprocesses, all contributing to loop closure. A critical point is to select organisms that can maintain high productivity in low gravity or high radiation levels, and which can rely, as nutrients, on materials naturally available on the Moon or Mars (partly or exclusively).

On Earth, the organisms used in biotechnology can be (environmental) natural or (gnotobiotic) synthetic communities, laboratory-selected axenic isolates, cultivars, or engineered organisms used in industrial and agricultural applications. Some of the microbial and plant biotech applications rely on metabolic engineering, the use of recombinant DNA techniques and/or heterologous expression, to tailor cell production towards the desired end products. Current space research has been focusing on a limited number of well-known organisms. For space BLSS applications in Europe, to our knowledge, only natural strains, communities, and cultivars have been shortlisted and investigated so far. On Earth, many bioprocesses (such as food processes) use well-characterized ‘industrial’ strains specifically selected to ‘fit for purpose’ (e.g., yeast for bread or beer production), which are robust strains that have been specifically evolved over many cycles and adapted for high productivity, in the specific engineered production conditions. Even genetically modified organisms (GMOs) are used in R&D or production outside Europe^59^. In addition, the use of plant biotechnology could be useful for modeling and for the definition of a space ideotype. Adaptive evolution or other bio-engineering tools could be useful to obtain suitable ‘space adapted’, and ‘ISRU-optimized’, strains/cultivars.

**Fig. 4:**
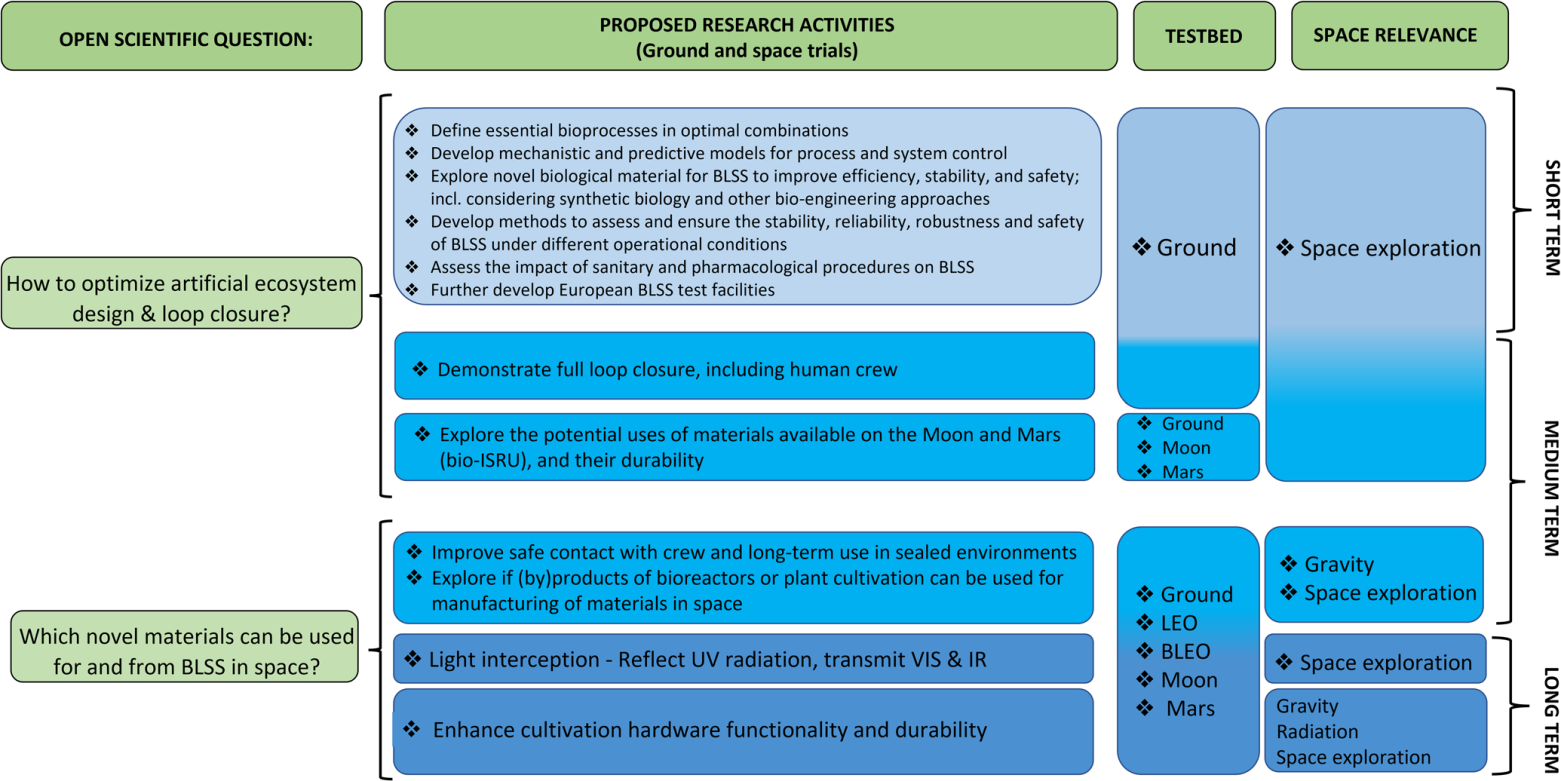
Summary of possible goals and sub-goals to realize a BLSS in the timeframe of 2022–2030 and beyond (short-term, 2022–2024; medium-term, 2024–2030; long-term, beyond 2030) using primarily the ISS platform. Other research platforms such as the ground, Moon, Mars, and BLEO are also included. BLEO is referred to long-term missions beyond LEO, also involving only traveling/transit without the permanence on a planetary station. They represent both the basis for the research on ISS and future research activities post-ISS (e.g., the GATEWAY orbiting the Moon).

To achieve the challenge of BLSS realization, two main issues need to be addressed, namely “how to optimize artificial ecosystem design and loop closure”, and eventually “which novel materials can be used for and from BLSS in space”. To address these issues, specific knowledge gaps need to be filled as reported in Fig. 4.

A key objective is to define the essential bioprocesses and biomaterials, as well as minimal and optimal combinations thereof, to reach maximum conversion efficiency and resource recovery. Discoveries and new insights into bioprocesses, nutrient cycles, and ecosystem functioning, should be adopted. Moreover, the application of a life cycle analysis approach may be useful for the analysis of the interconnections among compartments. A “biomanufacturing” approach based on in situ resource utilization and integration as described by Berliner et al. (2021)^60^ should be considered to achieve human-based space exploration.

Further, mechanistic knowledge models and predictive models for process and system control should be developed. Indeed, process and system control require (predictive) modeling, which in return requires a profound understanding of the elementary biological, physiological, and physical mechanisms. The potential of existing and future biomaterials and new biomaterials for BLSS should be explored more extensively, going beyond model species/cultivar/strains, and evaluating the usability of a variety of elements to identify the most suitable candidates. In bioprocesses, a point is to evaluate whether an artificial ecosystem can be fully built with separated, well-characterized axenic cultures or gnotobiotic communities (which is preferred for process control and product safety reasons); or if the use of complex or new natural, unidentified microbes, and communities is required/beneficial. In the latter case, a thorough assessment is required of how the use of unidentified communities, including their self-adaptation to the space conditions and self-evolution, will challenge/hamper the rational mechanistic design and control strategies for engineered BLSS, and the predictive evaluation of the performances and the risks. This approach could be considered to fulfill the following points:

- Increase the fitness of relevant organisms in specific environmental conditions.
- Improve the efficiency of existing bioprocesses.
- Enable new biological functions (e.g., production of new compounds, valorization of hard-to-recycle waste, utilization of materials available on the Moon and Mars).
- Explore the potential uses of materials available on planetary surfaces (e.g., Lunar and Martian regolith, Martian atmosphere) as inputs for BLSS (ISRU), to improve system sustainability.

For ISRU realization, the following issues should be considered:

- Develop methodologies and technologies for the use of planetary substrates as nutrient sources for BLSS (e.g., methods for leaching and extraction of nutrients for fertilizers).
- Develop methodologies and technologies for the use of planetary materials as physical support (e.g., as carrier materials or substrate for plant cultivation), also defining procedures and protocols to improve the physical and chemical properties (i.e., reduce toxicity) of Lunar and Martian regolith.
- Develop specific procedures for regolith amendment using wastes derived from the different BLSS compartments.
- Develop methodologies and technologies for the use of planetary materials as the structural material for the BLSS infrastructure and cultivation facility itself (skeleton, tanks, etc.). It should be assessed which components of BLSS can be manufactured in flight, e.g., via off-Earth 3D printing using materials available on the Lunar and Martian surfaces.
- Assess the durability of such materials (e.g., substrates) during prolonged use or reuse over multiple cultivation cycles.

Another gap is the development of methods and procedures to assess and ensure the stability, reliability, robustness, and safety of subsystems, and the complete BLSS under different operational conditions in the various mission scenarios.

Another point to be considered is the impact of sanitary and pharmacological countermeasures on circular BLSS to define rules for the inclusion or exclusion of certain waste streams. Although clearly secondary to its safety for the human host and treatment efficacy, the secretion and recalcitrance of a drug and its fate in a waste regenerative and circular food production system (and all its steps), should be assessed, and taken onboard as a selection criterion for drugs to be deployed in future space missions that, require regenerative life support^61^.

It goes without saying that there is an urgent need to further develop European BLSS facilities on Earth for long-duration, integrated testing, including all modules of BLSS (e.g., reactors, bioreactors, higher plants chambers, separators, purification processes) in combination with other habitat and crew activities (e.g., EVAs, medical-psychological-behavioral-acceptability aspects). These integrated test facilities must be modular, robust, and easy to reactivate and expand. They would be fundamental to demonstrate ideal loop closure, on Earth, at representative crew size and duration.

The possible introduction of novel biomaterials deserves a specific focus since it contains specific sub-gaps (Fig. 4). Microbial and plant biotechnology in space is a major step up from current Earth-based biotechnology and an additional incentive to explore novel materials for and from BLSS. Ideally, such novel materials should be recyclable in a closed loop. In this vision, it becomes fundamental to improve the biocompatibility of BLSS materials and products for safe contact with the crew and long-term use in sealed environments. In a circularity framework, it should be explored whether (waste) products of bioreactors or plant cultivation can be used for the ‘bioprinting’ of biomaterials in support of human tissue engineering as well as to produce biomaterials for (bio) fabrication and (bio) manufacturing onboard space stations^62,63^. Some examples of biomaterials are oils and lubricants, biofuels, bioplastics, and bio-inks for 3D printing (e.g., of biofilms). It is worth exploring whether novel biocompatible materials can be developed for light interception in photo-bioreactors and plant growth modules, to reflect UV radiation while transmitting visible and infrared radiation. Those materials would, ideally, be able to withstand large inside-outside pressure differences and prevent overheating but allow efficient thermal control (e.g., would dissipate most of the heat of the infrared radiation). Finally, the possibility to incorporate new and bio-interactive functionalities in materials (e.g., flexibility, transparency, surface tension control, biodegradability, resistance to sterilization, resistance to biofouling, sensing devices, etc.) should be evaluated to enhance cultivation hardware functionality and durability.

## Priorities for space programs and benefits for Earth and industrial relevance

Any BLSS for space application must be considered as a reduced-size mockup of a terrestrial ecological system. Any BLSS development is subject to an intrinsic and mandatory system-level evaluation. A BLSS for space must accomplish the basic needs of an advanced LSS provided by the NASA Baseline Values and Assumptions Document (BVAD)^64^. About 40–50 m^2^ of crops grown under high light intensities (>500 μmol m^−2^ s^−1^) would be needed to produce enough dietary calories and to supply all the O_2_ production and CO_2_ removal for one human. However, all BLSS studies so far have been ground-based, and the results have to be tested in space conditions^33^.

Priorities for space programs require improvements in the following areas of interest:

- The “microbe compartments”: for the efficient recycling of organic wastes and also for microbes’ utilization as amendments for Martian and Lunar regolith.
- The “plant compartment”: for the efficient cultivation of staple and non-staple crops (including polyculture) for resource regeneration and food production, as well as the possibility to achieve the *seed-to-seed* cycle to achieve independence from Earth supplies.
- The fine remote monitoring and control of environmental conditions and automatization of all the processes.
- The possible introduction of novel biomaterials.

**Fig. 5:**
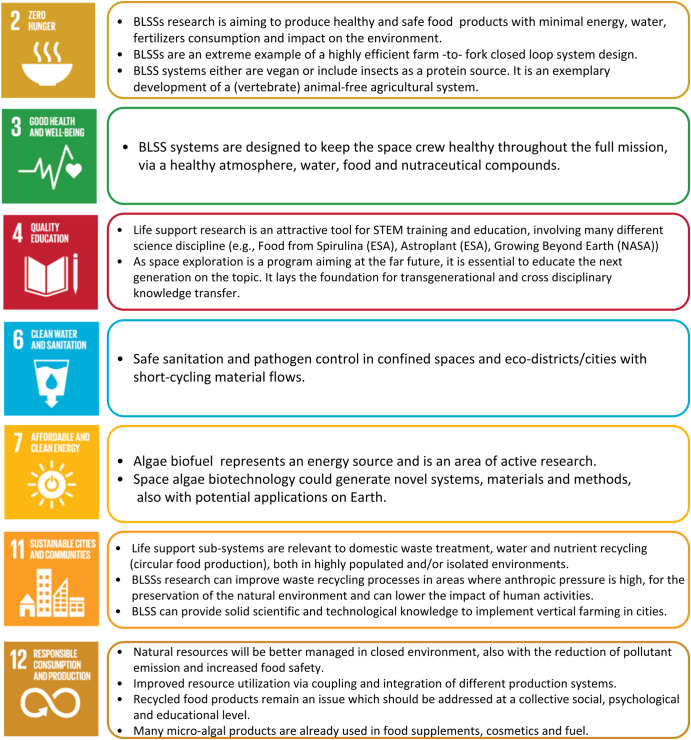
Schematic representation of how research goals to realize a BLSS have relevance to many Sustainable Development Goals (SDGs) of the EU. Therefore, space-oriented research can bring benefits for Earth sustainability targets.

These areas are indeed of interest also for Earth processes, since in BLSS the questions and problems dealt with are fundamentally identical to those that are addressed to achieve sustainability of processes in the management of our Earth ecosystems. It comes without saying that this type of knowledge development is of high importance for environmental engineering on Earth today, similar to the importance of Information and Communication Technologies (ICT) space programs for computer sciences in the sixties. Indeed, this kind of research is of benefit to the agri-food and health sectors on Earth, where it has already led to valuable applications such as improvements in automation and control in crop monitoring or freeze-dried foods and will continue bringing multiple benefits. Furthermore, it is perfectly in line with the circular economy and the New Green Deal policies of the EU Commission^65^. The BLSS research has a clear relevance to many sustainable development goals (SDGs) of the EU and particularly to SGS n. 2 (Zero Hunger), 3 (Good Health and Well-Being), 4 (Quality Education), 6 (Clean Water and Sanitation), 7 (Affordable and Clean Energy), 11 (Sustainable Cities and Communities) and 12 (Responsible Consumption and Production), as summarized in Fig. 5. The 17 goals cover social, economic, and environmental development challenges and each one has a set of targets, which are interconnected so that the success of one goal always involves addressing multiple other goals^66,67^. Just to mention a few, BLSS research supports precision agriculture, contributing to developing tools to improve crop monitoring to provide more valuable data to farmers and help them to improve yield and avoid food shortages (SDGs 2,6). It will be possible to improve the use of satellites to map the spread of diseases and public health emergencies (SDG 3), enable children to learn remotely and increase awareness on STEM education opportunities (SDG 4), and make big steps in water purification and resource regeneration to promote and increase recycled resource/products (SDG 12). Therefore, space is a great tool to help the community achieve the SDGs on Earth in a vision of a circular economy.

## Conclusions

The development of life support systems is a multi-disciplinary and multi-generational endeavor. Scientists and engineers of today are developing the systems of tomorrow, needed for the Moon and Mars missions of the following decades. The joint efforts of many disciplines spanning from microbiology to botany, from horticulture to system technology, and from cell biology to biotechnology, are leading to scientific and technological advances which are bringing immediate benefits also to Earth. Still, many gaps exist at the level of fundamental sciences and technological viewpoints to realize and integrate subsystems into a close BLSS. Any BLSS design will require a smart combination of multiple organisms and bioprocesses which can maintain high functioning efficiency in altered gravity or high radiation levels, and which could benefit from the use of resources available in situ.

To achieve the still numerous challenges, joint efforts are also needed to invest in the next generations of young STEM (Science Technology Engineering Mathematics) talents. Moreover, entrepreneurs should be involved early on. To do this, educational, training, and communication programs should be developed including:

- all relevant STEM disciplines: microbial/plant/animal/human biology, molecular biology, biochemistry, bioinformatics and biostatistics, bioscience engineering, environmental engineering, mathematics, modeling, control and automation, agricultural sciences and technology, food science and technology, design sciences, as well as cross- and interdisciplinary programs.
- all public types and ages: primary and secondary school projects, BSc and MSc teaching curricula and thesis projects, scientific and technical internships and visits, PhD and postdoc programs, summer schools, workshops, citizen science projects, scientific and general public conferences, etc.

The framework vision of such investigations is to achieve the target of going beyond the “simple” human survival in space to long-term space human habitability.

### Reporting summary

Further information on research design is available in the Nature Research Reporting Summary linked to this article.

### Supplementary information

Reporting Summary

## Data Availability

No data was generated throughout the manuscript.
